# Inefficiency of SIR models in forecasting COVID-19 epidemic: a case study of Isfahan

**DOI:** 10.1038/s41598-021-84055-6

**Published:** 2021-02-25

**Authors:** Shiva Moein, Niloofar Nickaeen, Amir Roointan, Niloofar Borhani, Zarifeh Heidary, Shaghayegh Haghjooy Javanmard, Jafar Ghaisari, Yousof Gheisari

**Affiliations:** 1 Regenerative Medicine Research Center, Isfahan University of Medical Sciences, Isfahan, 81746-73461 Iran; 2 Department of Electrical and Computer Engineering, Isfahan University of Technology, Isfahan, 84156-83111 Iran; 3 Department of Physiology, Applied Physiology Research Center, Isfahan Cardiovascular Research Institute, Isfahan University of Medical Sciences, Isfahan, Iran

**Keywords:** Viral infection, Computational models, Epidemiology

## Abstract

The multifaceted destructions caused by COVID-19 have been compared to that of World War II. What makes the situation even more complicated is the ambiguity about the duration and ultimate spread of the pandemic. It is especially critical for the governments, healthcare systems, and economic sectors to have an estimate of the future of this disaster. By using different mathematical approaches, including the classical susceptible-infected-recovered (SIR) model and its derivatives, many investigators have tried to predict the outbreak of COVID-19. In this study, we simulated the epidemic in Isfahan province of Iran for the period from Feb 14th to April 11th and also forecasted the remaining course with three scenarios that differed in terms of the stringency level of social distancing. Despite the prediction of disease course in short-term intervals, the constructed SIR model was unable to forecast the actual spread and pattern of epidemic in the long term. Remarkably, most of the published SIR models developed to predict COVID-19 for other communities, suffered from the same inconformity. The SIR models are based on assumptions that seem not to be true in the case of the COVID-19 epidemic. Hence, more sophisticated modeling strategies and detailed knowledge of the biomedical and epidemiological aspects of the disease are needed to forecast the pandemic.

## Introduction

The Coronavirus disease 2019 (COVID-19), has severely affected different aspects of human health all over the world. The high transmissibility rate and lack of efficient therapeutics have transformed the situation into a major challenge^1–3^. Ranking fifth according to the outbreak, announced the first cases on February 19, 2020. The pandemic quickly impacted most regions of the country with the highest rates in Tehran, Qom and Isfahan provinces^4,5^.

Virus epidemics have been known as one of the major health issues causing a high mortality rate in human communities throughout history. The Spanish flue emerged in 1918, caused 50 million deaths for just over 2 years^6^. Also, since the early reports of HIV infection in 1980, more than 32 million deaths have been reported around the world due to virus infection^7^. Tragically, not only these older global epidemics but also, the local spread of SARS, MERS, and Ebola viruses in recent years have not made countries ready for such crises. The lack of preparation for the outbreak of COVID-19 beside the high rate of transmissibility, unknown complications, and inappropriate medications led to a world-wide disaster paralyzing the health care systems even in developed countries. More importantly, the future course of the disease is not yet clearly known in many affected countries and the estimates of the ultimate spread of the epidemic are not coherent. This makes policy-making and planning for the required resources very difficult for the governments^8^. In this unknown situation, forecasting approaches are of particular importance.

Since the emergence of COVID-19, different mathematical modeling approaches have been employed to simulate the disease course^9^; the artificial intelligence-based models^10^ are interesting but as they depend on many learning steps, their validity can be questioned in the absence of sufficient training datasets. Day-level forecasting based on time-series data is another approach^11^ that simply follows previous patterns and cannot predict the trend of changes. Agent-based modeling is also a rational approach for predicting the disease course, which simulates the fellow of the individuals (agents) to calculate the disease spread in the community^12^. Nevertheless, such models rely on the population-level parameters such as rates of movements, distancing and virus infectivity parameters which are not yet known. Ordinary differential equation (ODE)-based models have been used for a long time to simulate the classical dynamics of epidemics^13^. This type of models was first proposed by Kermack and McKendrick in 1927 to simulate the transmission of infectious disease such as measles and rubella^14^. Such models assume susceptible (S), infective (I), and recovered (R) fractions in a close population and calculate the rate of changes in each fraction with ODEs^15,16^.

Over the past year, various simple and modified SIR models have been developed to predict the course of COVID-19; Anastassopoulou et al. provided a preliminary estimate of the fatality and recovery ratios for the population of Wuhan by a discrete-time SIR model^17^. Moreover, a model was developed by Giordano et al. that predicted the effect of different lockdown strategies on the epidemic in Italy. They considered different sub-groups regarding the stage and severity of the disease in the infected individuals^18^. Similarly, using an SEIR (Susceptible-Exposed-Infectious-Recovered) framework, Lin et al. predicted the effect of government policies and individual actions on the spread of the epidemic in China^19^. It should be noted that the modified SIR models require more complex data for the development, and due to the presence of little information and lack of reliable data regarding this newly emerged disease, the simple SIR model has been the choice of many investigators^20^.

Despite the simplicity and effectiveness of SIR-based models in forecasting a variety of other infectious diseases, their capability in the case of the COVID-19 pandemic is a matter of debate. In this regard, an SIR model was developed using the data of the first 3 months of COVID-19 spread in Isfahan province and the conformity and accuracy of the model was evaluated using the actual data in later months. Results of this study scrutinize the appropriateness of SIR models for the simulation and prediction of the COVID-19 epidemics.

## Material and methods

### SIR model

In the SIR models, the population is considered to be closed and the sum of susceptible, infective or recovered fractions denoted by $i\left( t \right)$, $s\left( t \right)$, and $r\left( t \right)$, respectively, is equal to 1 for each $t \ge 0$. The model is defined by the following set of ODEs:

1 $$\frac{ds}{{dt}} = - \lambda si,$$

2 $$\frac{di}{{dt}} = \lambda si - \gamma i,$$

3 $$\frac{dr}{{dt}} = \gamma i,$$

where $\lambda$ is the infection rate and $\gamma$ is the recovery rate. R_0_ is known as the basic reproduction number and defined as:

4 $$R_{0} = \frac{\lambda }{\gamma }.$$

R_0_ is an essential determinant of outbreaks and can be interpreted as the expected number of new cases directly caused by an infectious individual before the recovery.

All simulations were performed using MATLAB R2017a^21^.

### Epidemiological data

The data used in this study was provided by the medical care monitoring center (MCMC) of Isfahan University of Medical Sciences, Isfahan, Iran. This data consists of the daily hospitalized cases and deaths from all cities of Isfahan province except Kashan, Aran and Bidgol which are in the zone of Kashan University of Medical Sciences. The population of the studied region is approximately 4,632,000. Considering the low negative predictive value of PCR test for the diagnosis of COVID-19^22^, all patients admitted to hospitals with the clinical suspicion of the disease were considered as infected cases. This can describe the potential discrepancy between the actual cases assumed in this study and other reports that rely on the molecular diagnosis of the infection.

## Results

### Using the epidemiologic data for the first 3 months, an SIR-based model was constructed to predict the disease course

In the SIR model, $r\left( t \right) + i\left( t \right){ }$ determines all the individuals who are no longer susceptible. The term can be interpreted as the accumulative number of new cases in each time-point that can be recovered, died or are still infectious due to the course of disease. According to recent studies, 30% of infected individuals are asymptomatic that are not identified in the population unless in active case finding programs^23^. Out of the remaining infected cases that manifest the disease, 70% show mild to moderate symptoms and are treated in out-patient settings without any need to hospitalization. whereas, 30% of symptomatic cases have severe manifestations and must be admitted to hospitals for a while during the infection^24,25^. Hence, the cumulative number of new hospital-admitted cases can be estimated from Eq. (5):

5 $$Cumulative{ }\,hospitalized\,{ }patients{ } = { }0.21{ }\left( {r\left( t \right) + i\left( t \right)} \right).$$

In addition, the number of cumulative deaths is assumed as:

6 $$Cumulative\, death = K \times \left( {r\left( t \right) + i\left( t \right)} \right).$$

Although a mortality rate of 2–3% is reported for COVID-19 patients^26^, the actual rate depends on a variety of parameters including the average age of the population and the capacity of healthcare systems. In this regard, the parameter $K$ is determined for each population individually based on the reported mortality rates.

**Figure 1:**
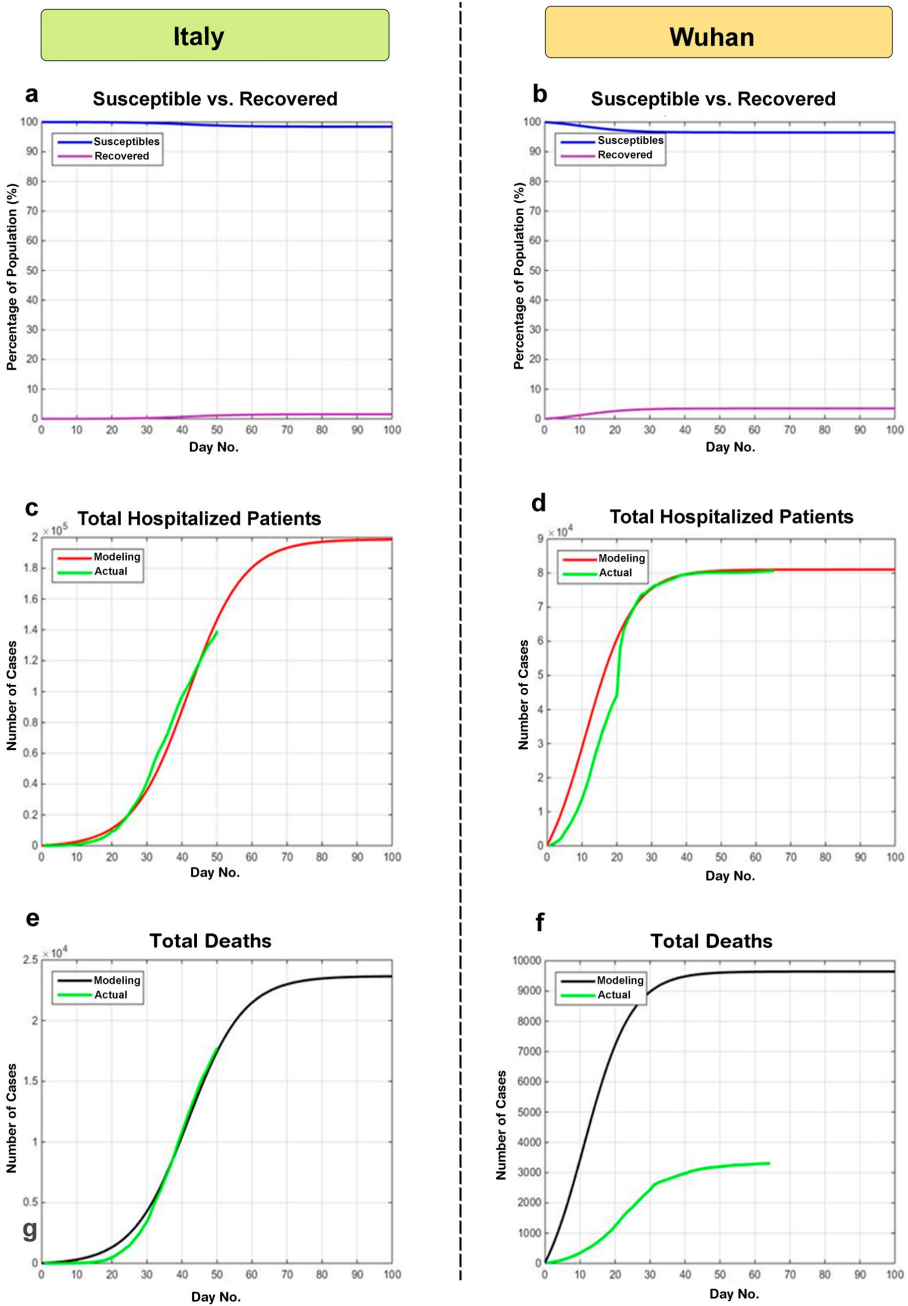
Modeling COVID-19 epidemic in Wuhan and Italy. To provide a rough estimation of the epidemic dynamics, the model was first generated with the reported data of Wuhan and Italy. (**a**,**b**) Epidemic final size. (**c**,**d**) Cumulative hospitalized cases. (**e**,**f**) Cumulative deaths.

To estimate the R_0_ value for the COVID-19 epidemic, the model was first fitted to the cumulative new cases data reported until April 10th, 2020 from Wuhan and Italy and the epidemic final size (Fig. 1a,b), Cumulative hospitalized cases (Fig. 1c,d), and the Cumulative deaths (Fig. 1e,f) were predicted. Then the model was fine-tuned based on Isfahan statistics.

An R_0_ of 1.0078 could best describe the data of Italy. Also, the curves of Wuhan could be appropriately simulated when R_0_ was assumed 1.0146 (Fig. 1d). Notably, the number of new hospital-admitted cases in Italy showed a decline in days 40–50 which could be attributed to the strict social distancing policies imposed by the government (Fig. 1c). To fit the model to the mortality data, parameter $K$ (Eq. 6) was set at 0.025 for Italy. Notably, when the same value was considered for Wuhan, the simulation curve was far higher than the reported official data (Fig. 1f). In agreement with this observation, the Chinese authorities have later declared the inaccuracy of the initial reports of deaths in Wuhan and boosted the number by 50%^27^.

The simulations for Wuhan and Italy allowed starting the modeling of the epidemic in Isfahan with a rough estimation of R_0_ value. Although, each outbreak is described with a unique R_0_ in classical SIR models^28,29^, we decided to use a set of R_0_ values for different time intervals to account for the variations of the community behavior and inconsistency of social distancing regulations. At the time of model construction, the actual epidemiological data were available for the episode from Feb 14th to April 11th. The model had the best fit to the actual data when four different R_0_ values were considered. Since the changes in social distancing are reflected in the hospital admission rates with a time delay of about 2 weeks^30^, the four R_0_ values were corroborated with community behavior variations as represented by city traffic reports (data not shown).

To forecast the epidemic in this province, three scenarios based on the strictness level of social distancing were assumed and different R_0_ values were considered in each case (Table 1). From Feb 14th to April 11th, the highest value of R_0_ was 1.0165 (R_01_) which is attributed to the beginning of the epidemic when people were not aware of the outbreak and thus, no restriction was imposed. In the “bad scenario”, R_0_ again reached the same value after a transitional step. In addition, during the mentioned period, the smallest value of R_0_ was 1.0040 (R_03_). Hence, considering the practical issues, it is assumed that R_0_ may again reach to a value as small as 1.0050 in the “good scenario”. Also, an intermediate value of 1.0095 was considered for the “feasible scenario”.

**Table 1:** R_0_ values used in the SIR model.

Simulating actual data				Forecasting		
R_01_ 02/14–02/28	R_02_ 02/29–03/14	R_03_ 03/15–03/28	R_04_ 03/29–04/12	Scenarios	R_05_ 04/13–04/27	R_06_ ≥ 04/28
1.0165	1.0060	1.0040	1.0085	Good	1.0055	1.0050
				Feasible	1.0090	1.0095
				Bad	1.0110	1.0165

**Figure 2:**
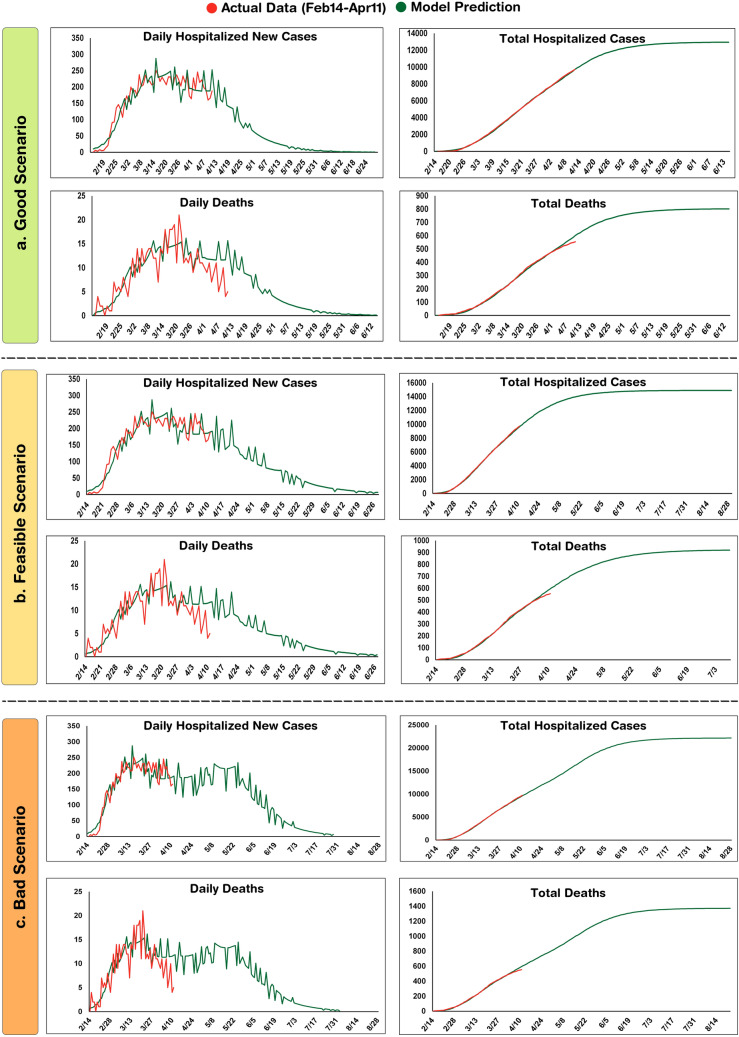
Modeling COVID-19 epidemic in Isfahan province using the first 3 month data. To forecast the epidemic, Good (**a**), Feasible (**b**) and Bad (**c**) Scenarios are assumed.

In the good scenario achieved by strict social distancing, the SIR model predicted that about 13,000 cases would be cumulatively hospitalized by the end of the epidemic and the total number of deaths would reach 800 cases. In this scenario, the curve of cumulative cases approaches a plateau on May 4 and by June 10, only 0–2 new cases would be admitted to hospitals on a daily basis (Fig. 2a).

The feasible scenario was attributed to closing schools, universities, cultural centers and limiting social gatherings and businesses that are not critical. In this scenario, the model predicted that total hospitalized cases and deaths would reach about 15,000 and 920, respectively. Also, the curve of cumulative cases would approach a plateau on May 24 and by July 18, it predicted that only 0–2 new cases would be admitted to hospitals on a daily basis (Fig. 2b). In the bad scenario, a situation was assumed in which the community is back to the routine life style. In this scenario, 22,000 cases would be totally admitted to the hospitals and the number of victims would be more than 1300. The model also forecasted that a second peak is unlikely and the epidemic would last up to mid-August (Fig. 2c). The results of the three scenarios are summarized in Table 2.

**Table 2:** Predicted outcomes of the three proposed scenarios.

Scenarios	Final epidemic size^a^	Total deaths	Total hospitalizations	Cessation date^b^
Good	1.40%	803	12,969	Jun 10, 2020
Feasible	1.50%	922	14,902	July 18, 2020
Bad	2%	1373	22,175	Aug 15, 2020

^a^The proportion of the community that becomes affected by the end of the epidemic.

^b^Daily hospitalized new cases ≤ 2.

### Evaluation of model predictions with actual epidemiologic data after 9 months

**Figure 3:**
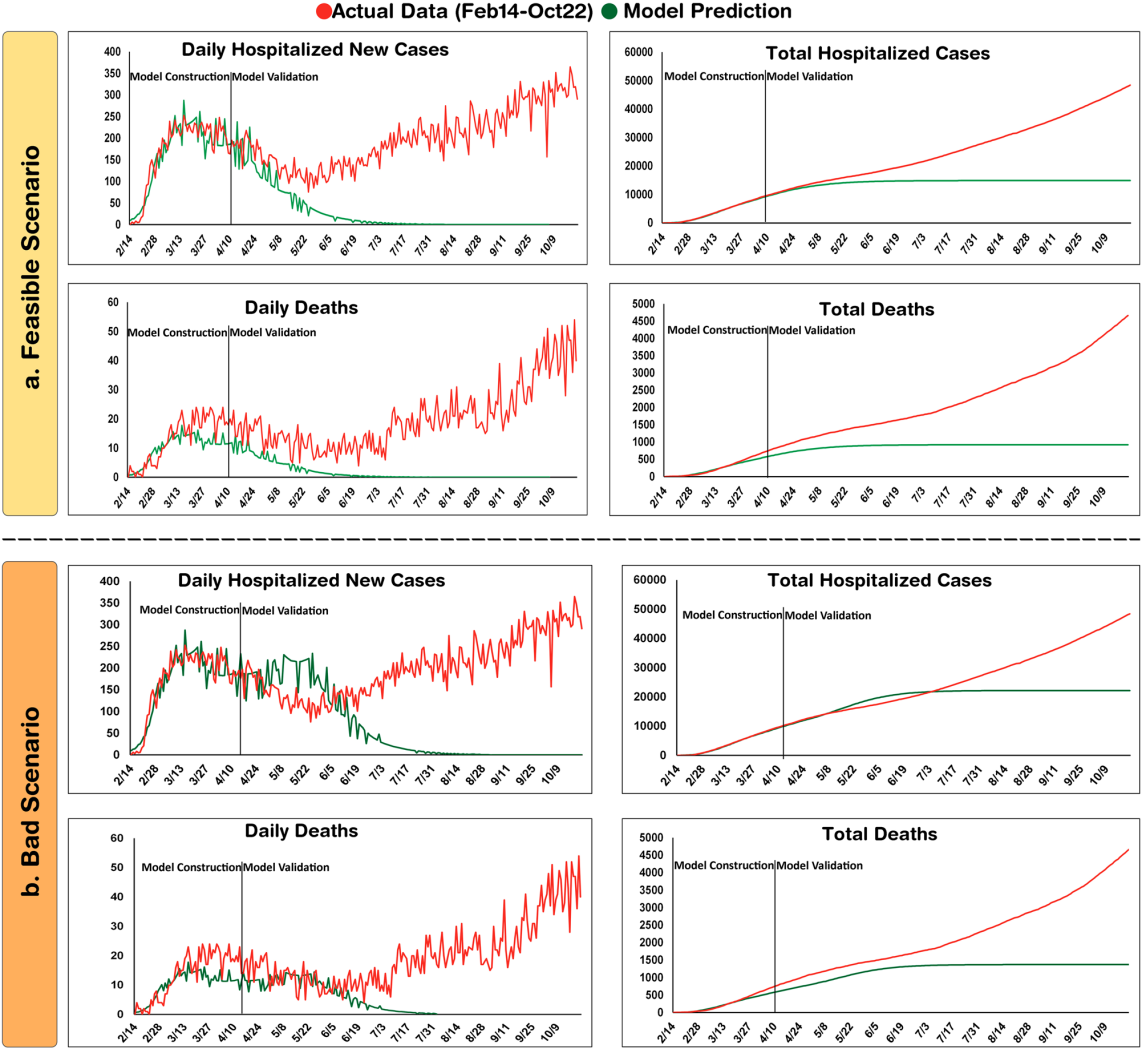
Evaluation of model predictions after 9 months. Actual data until October 22 are demonstrated in line with model predictions in Feasible (**a**) and Bad (**b**) scenarios. In each graph, the time-intervals for model construction and validation are indicated.

The prediction patterns in the three scenarios were evaluated by their conformity to actual data over the 9 months since the start of the epidemic. Although the good-scenario predictions were far from the pattern of epidemic (data not shown), the trend of feasible scenario could appropriately match with the daily hospitalized cases until mid-June (Fig. 3a). However, a new long-lasting huger peak was appeared at the beginning of July which was not anticipated even in the bad scenario, leading to the increase of total infections to 43,293 until October 22 (Fig. 3b). Although the model could forecast disease course patterns in the short term, the predicted values were far from the actual data in later time intervals and the constructed model was unsuccessful at long term prediction of epidemic course.

## Discussion

COVID-19 pandemic is emerged as a massive health burden all over the world. Mathematical modeling is a useful approach for the prediction of disease dynamics to take best decisions. Currently, various mathematical approaches have been developed for modeling the COVID-19 pandemic which mostly depend on a number of parameters for the simulation^31^. Due to the lack of access to adequate information about this newly emerged pandemic, simple strategies such as SIR-based models have been implemented by several investigators. However, their accuracy and suitability for prediction of the COVID-19 disease course is a matter of debate. In this study, using the data of the first 3 months of the COVID-19 epidemic in Isfahan, an SIR model was developed and the outcome was predicted with three scenarios that differ in terms of social distancing stringency. Then, the constructed model was evaluated for conforming the predicted values to the actual data in the six consecutive months. Although the model could appropriately simulate the patterns until the mid-June, it was unable to predict the second peak of epidemic emerged in July.

The inability of our model to predict COVID-19 pandemic in Isfahan motivated us to assess the conformity of the predictions of SIR models developed by other investigators to the actual data of different regions around the world. Although the SIR model developed by Ahmetolan et al. could predict the course of disease in the countries with controlled epidemics, such as China, Korea, and Singapore, it was unable to forecast the larger peak mainly started at the late summer in many countries located in the northern hemisphere^32^. Similarly, the modified SIR model constructed by Cooper et al., which was manually adjusted to fit actual data in a period of time^33^, was unable to predict the next surges, and the estimates of infected populations were also far from reality. For instance, the model predicted 330,000 infections at the end of September in India, but it actually turned to be around 900,000^34^. Morover, the extended SIR model of Wangping and colleagues estimated that the epidemic in Italy would be ended in August with a total number of 182,051 infected cases. Nevertheless, according to actual data, a second peak started in October with more than 700,000 total infected cases^35^. Similar inconsistencies between predictions of SIR-based models and actual data for the COVID-19 epidemic could be observed in other studies^28,36–38^.

The failure of SIR-based models to forecast the COVID-19 pandemic can be described by a variety of reasons. These over-simplified models ignore the factors that have a great effect on the course of disease. For instance, various studies demonstrate that air pollution is a main reason for the observed variations in disease contagion^39,40^. The other issue affecting the spread of virus is the behavioral changes considerably related to the social and cultural context of the population^41^. As mostly a single R_0_ value is considered in the SIR models, the unexpected changes in the social behaviors of the population are missed and the model would be unable to follow the emerging alterations. Indeed, ceremonies and national gatherings have a great impact on disease spread. For instance, a religious meeting in Malaysia, held on February 27 to March 3 is supposed to be the source of virus spread in India and Pakistan^42^. Similar social events in Isfahan, at least partly, may describe the unpredicted peak starting from July.

Another explanation for the failure of SIR-based models to predict the COVID-19 epidemics is that the modeling is based on the assumptions that are not necessarily true; the population is considered closed in the SIR models, whereas, complete isolation was not followed in most regions, making them vulnerable to changes in the neighboring communities. In addition, the recovered individuals are assumed as immunized in the SIR models, which are no longer susceptible. This assumption contrasts with new findings suggesting there is a possibility of the reactivation of the virus or reinfection of previously infected individuals^43–45^. Indeed, a recent study indicates that the longevity of neutralizing antibodies in infected individuals is only around 50–60 days ^46^.

## Conclusion

Taken together, the COVID-19 pandemic features are not coherent with the SIR modeling framework and the dynamics of this outbreak is under the influence of various parameters for most of which quantitative information is not yet available. More sophisticated modeling approaches in line with more precise epidemiological and biomedical data are urgently required to make the pandemic forecasting feasible.

## References

[1] Liu Y, Gayle AA, Wilder-Smith A, Rocklöv J (2020). The reproductive number of COVID-19 is higher compared to SARS coronavirus. J. Travel Med..

[2] Ferguson, N. *et al.* Impact of non-pharmaceutical interventions (NPIs) to reduce COVID19 mortality and healthcare demand. *Imperial Coll. Lond.*10.25561/77482 (2020).

[3] Yuan J, Li M, Lv G, Lu ZK (2020). Monitoring transmissibility and mortality of COVID-19 in Europe. Int. J. Infect. Dis..

[4] Aminian A, Safari S, Razeghian-Jahromi A, Ghorbani M, Delaney CP (2020). COVID-19 outbreak and surgical practice: Unexpected fatality in perioperative period. Ann. Surg..

[5] Abdi M (2020). Coronavirus disease 2019 (COVID-19) outbreak in Iran: Actions and problems. Infect. Control Hosp. Epidemiol..

[6] Taubenberger JK, Morens DM (2006). 1918 Influenza: The mother of all pandemics. Rev. Biomed..

[7] UNAIDS. https://www.unaids.org/en/resources/fact-sheet.

[8] Gates B (2020). Responding to Covid-19—A once-in-a-century pandemic?. N. Engl. J. Med..

[9] Rabajante, J. F. Insights from early mathematical models of 2019-nCoV acute respiratory disease (COVID-19) dynamics. *arXiv preprint *arXiv:2002.05296 (2020).

[10] Hu, Z., Ge, Q., Jin, L. & Xiong, M. Artificial intelligence forecasting of covid-19 in china. *arXiv preprint *arXiv:2002.07112 (2020). 10.3389/frai.2020.00041 PMC7861333 33733158

[11] Elmousalami, H. H. & Hassanien, A. E. Day level forecasting for coronavirus disease (COVID-19) spread: Analysis, modeling and recommendations. *arXiv preprint *arXiv:2003.07778 (2020).

[12] Kim Y, Ryu H, Lee S (2018). Agent-based modeling for super-spreading events: A case study of MERS-CoV transmission dynamics in the Republic of Korea. Int. J. Environ. Res. Public Health.

[13] Satsuma J, Willox R, Ramani A, Grammaticos B, Carstea A (2004). Extending the SIR epidemic model. Phys. A.

[14] Bacaër, N. In *A Short History of Mathematical Population Dynamics* (ed Nicolas, B.) 89–96 (Springer, London, 2011).

[15] Zhou Y, Ma Z, Brauer F (2004). A discrete epidemic model for SARS transmission and control in China. Math. Comput. Model..

[16] Britton T (2010). Stochastic epidemic models: A survey. Math. Biosci..

[17] Anastassopoulou C, Russo L, Tsakris A, Siettos C (2020). Data-based analysis, modelling and forecasting of the COVID-19 outbreak. PLoS ONE.

[18] Giordano G (2020). Modelling the COVID-19 epidemic and implementation of population-wide interventions in Italy. Nat. Med..

[19] Lin Q (2020). A conceptual model for the coronavirus disease 2019 (COVID-19) outbreak in Wuhan, China with individual reaction and governmental action. Int. J. Infect. Dis..

[20] Roda WC, Varughese MB, Han D, Li MY (2020). Why is it difficult to accurately predict the COVID-19 epidemic?. Infect. Dis. Model..

[21] Ben-David U (2018). Genetic and transcriptional evolution alters cancer cell line drug response. Nature.

[22] Kokkinakis I, Selby K, Favrat B, Genton B, Cornuz J (2020). Covid-19 diagnosis: Clinical recommendations and performance of nasopharyngeal swab-PCR. Revue medicale suisse.

[23] Wang Y (2020). Clinical outcome of 55 asymptomatic cases at the time of hospital admission infected with SARS-Coronavirus-2 in Shenzhen, China. J. Infect. Dis..

[24] Lauer SA (2020). The incubation period of coronavirus disease 2019 (COVID-19) from publicly reported confirmed cases: Estimation and application. Ann. Intern. Med..

[25] Liu Y (2020). Viral dynamics in mild and severe cases of COVID-19. Lancet Infect. Dis..

[26] Wu Z, McGoogan JM (2020). Characteristics of and important lessons from the coronavirus disease 2019 (COVID-19) outbreak in China: Summary of a report of 72,314 cases from the Chinese Center for Disease Control and Prevention. JAMA.

[27] Science, L. https://www.livescience.com/wuhan-coronavirus-death-toll-revised.html.

[28] Zareie B, Roshani A, Mansournia MA, Rasouli MA, Moradi G (2020). A model for COVID-19 prediction in Iran based on China parameters. medRxiv..

[29] Calafiore, G. C., Novara, C. & Possieri, C. A Modified SIR Model for the COVID-19 Contagion in Italy. *arXiv preprint *arXiv:2003.07778 (2020). 10.1016/j.arcontrol.2020.10.005 PMC7587010 33132739

[30] Coronavirus disease 2019 (COVID-19) pandemic: increased transmission in the EU/EEA and the UK—seventh update. https://www.ecdc.europa.eu/sites/default/files/documents/RRA-seventh-update-Outbreak-of-coronavirus-disease-COVID-19.pdf.

[31] Tolles J, Luong T (2020). Modeling epidemics with compartmental models. JAMA.

[32] Ahmetolan S, Bilge AH, Demirci A, Peker-Dobie A, Ergonul O (2020). What can we estimate from fatality and infectious case data using the susceptible-infected-removed (SIR) model? A case study of Covid-19 pandemic. Front. Med. (Lausanne).

[33] Cooper I, Mondal A, Antonopoulos CG (2020). Dynamic tracking with model-based forecasting for the spread of the COVID-19 pandemic. Chaos Solitons Fractals.

[34] Coronavirus, w. https://www.worldometers.info/coronavirus/.

[35] Wangping J (2020). Extended SIR prediction of the epidemics trend of COVID-19 in Italy and compared with Hunan, China. Front. Med. (Lausanne)..

[36] Bastos, S. B. & Cajueiro, D. O. Modeling and forecasting the early evolution of the Covid-19 pandemic in Brazil. *arXiv preprint * arXiv:2003.14288 (2020). 10.1038/s41598-020-76257-1 PMC7655855 33173127

[37] Cintra HPC, Fontinele FN (2020). Estimative of real number of infections by COVID-19 in Brazil and possible scenarios. Infect. Dis. Model..

[38] Guirao A (2020). The Covid-19 outbreak in Spain. A simple dynamics model, some lessons, and a theoretical framework for control response. Infect. Dis. Model..

[39] Fattorini D, Regoli F (2020). Role of the chronic air pollution levels in the Covid-19 outbreak risk in Italy. Environ. Pollut..

[40] Comunian S, Dongo D, Milani C, Palestini P (2020). Air pollution and Covid-19: The role of particulate matter in the spread and increase of Covid-19's morbidity and mortality. Int. J. Environ. Res. Public Health.

[41] Bavel JJV (2020). Using social and behavioural science to support COVID-19 pandemic response. Nat. Hum. Behav..

[42] Quadri SA (2020). COVID-19 and religious congregations: Implications for spread of novel pathogens. Int. J. Infect. Dis..

[43] Alizargar J (2020). Risk of reactivation or reinfection of novel coronavirus (COVID-19). J. Formos Med. Assoc..

[44] Hoang VT, Dao TL, Gautret P (2020). Recurrence of positive SARS-CoV-2 in patients recovered from COVID-19. J. Med. Virol..

[45] Gousseff M (2020). Clinical recurrences of COVID-19 symptoms after recovery: Viral relapse, reinfection or inflammatory rebound?. J. Infect..

[46] Seow J (2020). Longitudinal observation and decline of neutralizing antibody responses in the three months following SARS-CoV-2 infection in humans. Nat. Microbiol..

